# Yohimbine Alleviates Oxidative Stress and Suppresses Aerobic Cysteine Metabolism Elevated in the Rat Liver of High-Fat Diet-Fed Rats

**DOI:** 10.3390/molecules28052025

**Published:** 2023-02-21

**Authors:** Małgorzata Iciek, Magdalena Górny, Magdalena Kotańska, Anna Bilska-Wilkosz, Marta Kaczor-Kamińska, Jacek Zagajewski

**Affiliations:** 1 Chair of Medical Biochemistry, Faculty of Medicine, Jagiellonian University Medical College, Kopernika 7, 31-034 Cracow, Poland; 2 Department of Pharmacological Screening, Faculty of Pharmacy, Jagiellonian University Medical College, Medyczna 9, 30-688 Cracow, Poland

**Keywords:** yohimbine, hydrogen sulfide, sulfane sulfur, cysteine

## Abstract

Yohimbine is a small indole alkaloid derived from the bark of the yohimbe tree with documented biological activity, including anti-inflammatory, erectile dysfunction relieving, and fat-burning properties. Hydrogen sulfide (H_2_S) and sulfane sulfur-containing compounds are regarded as important molecules in redox regulation and are involved in many physiological processes. Recently, their role in the pathophysiology of obesity and obesity-induced liver injury was also reported. The aim of the present study was to verify whether the mechanism of biological activity of yohimbine is related to reactive sulfur species formed during cysteine catabolism. We tested the effect of yohimbine at doses of 2 and 5 mg/kg/day administered for 30 days on aerobic and anaerobic catabolism of cysteine and oxidative processes in the liver of high-fat diet (HFD)-induced obese rats. Our study revealed that HFD resulted in a decrease in cysteine and sulfane sulfur levels in the liver, while sulfates were elevated. In the liver of obese rats, rhodanese expression was diminished while lipid peroxidation increased. Yohimbine did not influence sulfane sulfur and thiol levels in the liver of obese rats, however, this alkaloid at a dose of 5 mg decreased sulfates to the control level and induced expression of rhodanese. Moreover, it diminished hepatic lipid peroxidation. It can be concluded that HFD attenuates anaerobic and enhances aerobic cysteine catabolism and induces lipid peroxidation in the rat liver. Yohimbine at a dose of 5 mg/kg can alleviate oxidative stress and reduce elevated concentrations of sulfate probably by the induction of TST expression.

## 1. Introduction

Yohimbine is a small indole alkaloid derived from the bark of the yohimbe tree (Pausinystalia yohimbe) native to Central and West Africa, Cameroon, and Congo. It is an α_2_-adrenoceptor antagonist, and it has been documented to possess pharmacological activity. In Africa, it is traditionally used for fever, cough, heart disease, and atherosclerosis. It has been documented that this alkaloid is able to lower blood pressure and possesses anti-inflammatory and immunosuppressive properties [1,2]. The most well-known and widespread use of yohimbine is related to relieving erectile dysfunction and improving sexual stimulation [3]. It is probably connected with the inhibition of the α_2_ adrenergic receptors in the corpus cavernosum. Studies on animals revealed that yohimbine had a remarkably positive effect on sexual performance [4]. Due to its ability to selectively block α_2_-adrenoceptors in the brain, yohimbine can increase the release of norepinephrine and dopamine and improve feelings, thus, it is studied as a potential antidepressant [5]. Finally, yohimbine has been shown to induce fat loss and due to its presumed lipolytic properties, it is used for fast weight loss and bodybuilding [6,7].

**Figure 1:**
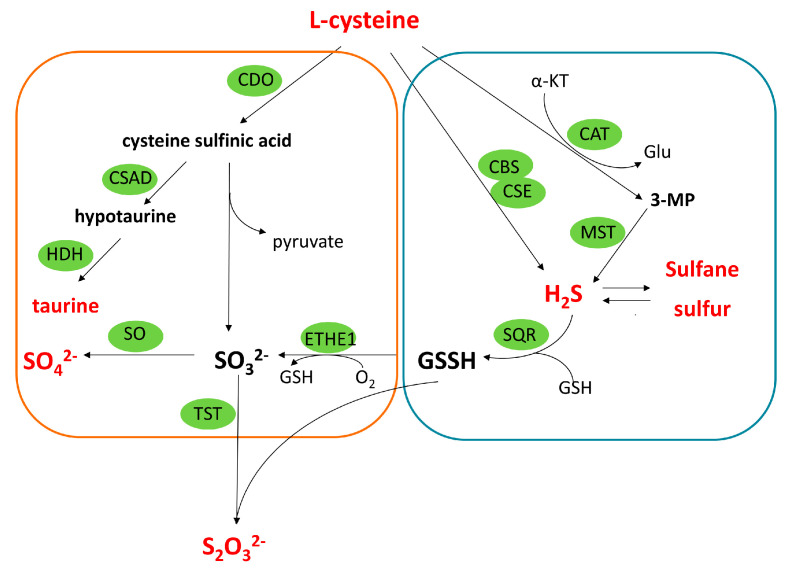
Aerobic (orange frame) and anaerobic (blue frame) catabolism of cysteine. L-cysteine is metabolized by the aerobic pathway in the reaction catalyzed by cysteine dioxygenase (CDO) to cysteine sulfinic acid, which can undergo decarboxylation catalyzed by cysteine sulfinic acid decarboxylase (CSAD) leading to hypotaurine formation. The latter compound is easily oxidized by hypotaurine dehydrogenase (HDH) to taurine. Cysteine sulfinic acid can also be metabolized via transamination to yield pyruvate and sulfite and the latter compound is oxidized by sulfite oxidase (SO) to sulfate. Taurine and sulfate are regarded as end products of aerobic cysteine catabolism. L-cysteine during its anaerobic transformations is also the main substrate for production of H_2_S and sulfane sulfur. H_2_S is synthetized from L-cysteine in reactions catalyzed by cystathionine β-synthase (CBS) and cystathionine γ-lyase (CSE). L-cysteine can also be transformed by cysteine aminotransferase (CAT) to 3-mercaptopyruvate (3-MP), which is a substrate for 3-mercaptopyruvate sulfurtransferase (MST). In biological conditions, H_2_S coexists in balance with a pool of reactive sulfane sulfur. This term covers bound sulfane sulfur (mainly persulfides and polysulfides) as well as other compounds containing reactive sulfur atoms, including elemental sulfur and polythionates. During the mitochondrial catabolism of H_2_S, thiosulfate and sulfates are formed as its end products in reactions catalyzed by sulfide quinone oxidoreductase (SQR), persulfide dioxygenase (ETHE1) and rhodanese (TST). During this catabolism, sulfane sulfur-containing persulfides are formed which underlines a close relationship between H_2_S and sulfane sulfur.

Obesity induced by improper nutrition, including a high-fat diet (HFD) is a widespread problem in developed countries. A common complication of obesity is non-alcoholic fatty liver disease characterized by chronic inflammation, oxidative stress, and steatosis. Many studies indicate that hydrogen sulfide (H_2_S), regarded as an important gaseous signaling molecule, plays an important role in the pathophysiology of obesity and obesity-induced liver injury [8]. H_2_S is synthesized endogenously mainly from L-cysteine. Enzymes involved in this process include cystathionine synthase (CBS), cystathionine γ-lyase (CSE), and 3-mercaptopyruvate sulfur transferase (MST) (Figure 1). CBS and CSE are cytosolic pyridoxal-dependent enzymes, while MST is localized mainly in mitochondria. In the nervous system, CBS and MST play a dominant role in the synthesis of H_2_S, while in the liver CSE and MST are the main H_2_S-producing enzymes [8,9,10]. H_2_S coexists in balance with a pool of reactive sulfane sulfur. This term refers to compounds containing a sulfur atom covalently bound to another sulfur atom. Sulfane sulfur possesses a high reactivity and is easily transferred to appropriate acceptors, such as SO_3_^2−^ or RS^−^, forming thiosulfate (S_2_O_3_^2−^) and persulfides RSS^−^, respectively. Sulfane sulfur-containing compounds include mainly persulfides and polysulfides as well as elemental sulfur and polythionates [11,12]. Compounds with sulfane sulfur can easily release H_2_S under reducing conditions. There is a close relationship between H_2_S and sulfane sulfur compounds, namely both of these reactive sulfur species are responsible for biological effects and both CSE and MST are involved in their formation. Due to the toxicity of high concentrations of H_2_S [13], it is important to maintain a balance between endogenous H_2_S synthesis, its storage in the form of sulfane sulfur, and H_2_S catabolism, thanks to which its concentration in cells can be kept in a low physiological range. Catabolism of H_2_S takes place in the mitochondria with the participation of sulfide quinone oxidoreductase (SQR), persulfide dioxygenase (ETHE1), and rhodanese (TST). The main end products of H_2_S catabolism include thiosulfate and inorganic sulfate anions. Sulfate and taurine are also regarded as the main products of aerobic cysteine catabolism (Figure 1). During the mitochondrial catabolism of H_2_S, sulfane sulfur-containing persulfides of SQRSSH and GSSH are formed which underlines a strict relationship between H_2_S and persulfides bearing sulfane sulfur.

The impaired endogenous H_2_S synthesis has been reported to be associated with obesity. It has been demonstrated that exogenous H_2_S or its donors can alleviate liver injury induced by HFD [14,15,16]. On the other hand, studies by Yang et al. demonstrated that exogenously applied H_2_S promoted fat accumulation in fruit flies while HFD-induced fat build-up was lost in CSE-deficient mice [17]. Moreover, a recent study by Comas et al. revealed that serum H_2_S concentration in morbid obesity patients was increased when compared to lean controls [18]. All these facts confirmed that H_2_S was involved in the pathogenesis of obesity and obesity-induced liver failure, however, the studies analyzed mainly the H_2_S concentration and expression of H_2_S-synthesizing enzymes. Sulfane sulfur compounds and sulfates have not been studied in this context.

As the biological activity of yohimbine, especially the regulation of metabolism related to obesity and its role in the treatment of erectile dysfunction, is similar to the well-documented properties of H_2_S, it seems that the mechanism of yohimbine action can be at least partially associated with the regulatory properties of reactive sulfur species, including H_2_S and sulfane sulfur. So far, no research has been conducted to clarify this relationship. Therefore, the aim of the present study was to investigate the effect of yohimbine on the level of H_2_S and sulfane sulfur, and on the activity and expression of enzymes involved in their synthesis in the liver of HFD-induced obese rats. Moreover, the levels of sulfate and thiols (cysteine and glutathione), and the concentration of malondialdehyde (MDA), as a measure of lipid peroxidation, were assayed. We hope that our study will shed new light on the biological potential of yohimbine in the context of HFD-induced liver injury, especially in relation to anaerobic and aerobic cysteine metabolism.

## 2. Results

### 2.1. The Effect of HFD and Yohimbine Treatment on the Changes in Body Weight

The mean initial body weight of all 24 young rats was 153.6 ± 3.8 g. Six rats were fed with standard food, while eighteen rats were fed with HFD. After 10 weeks, the weight gain in the control group was 206.7 ± 3.5 g, and in HFD-fed groups, the weight gain was higher (256.8 ± 4.8 g) and this increase was statistically significant (Table 1). During 30 days of the experiment, the weight gain in HFD-fed rats (O) was elevated when compared with control animals (C). Treatment with yohimbine at a dose of 5 mg/kg/day for 30 days caused significantly lowered weight gain when compared to the O group. Yohimbine at a dose of 2 mg/kg/day also diminished the weight gain but the effect was weaker (Table 1).

### 2.2. The Effect of HFD and Yohimbine Treatment on the Level of Free Sulfide, Sulfane Sulfur, and Bound Sulfane Sulfur in the Rat Liver

**Figure 2:**
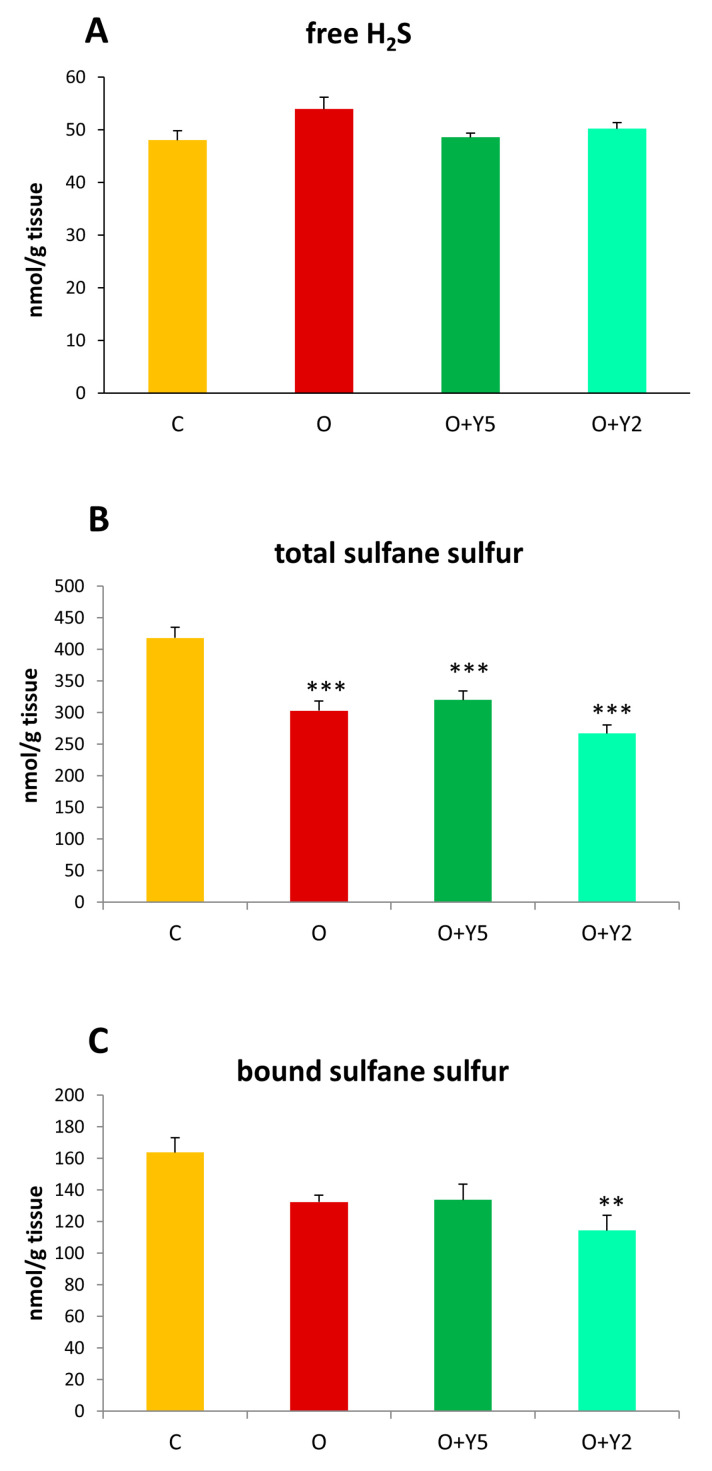
Concentrations of the free H_2_S (**A**), total sulfane sulfur (**B**), and bound sulfane sulfur (**C**) in the livers of rats. C—control rats fed a standard diet; O—rats fed with HFD; O + Y5—rats fed with HFD and treated intraperitoneally (i.p.) with yohimbine at a dose of 5 mg/kg/day for 30 days; O + Y2—rats fed with HFD and treated intraperitoneally (i.p.) with yohimbine at a dose of 2 mg/kg/day. Data are presented as the mean ± SEM, *n* = 6 per group. Statistical analysis was performed using a one-way ANOVA; symbols indicate significance of differences according to the Tukey post hoc test, ** *p* < 0.01, *** *p* < 0.001 vs. group C.

The level of free sulfide (H_2_S) did not differ significantly between the studied groups, however, a slight but statistically non-significant increase in H_2_S level could be seen in the liver of obese rats without yohimbine treatment when compared to healthy animals (Figure 2A).The level of the total pool of sulfane sulfur was significantly decreased in the liver of HFD-induced obese rats when compared to the liver of healthy animals. Treatment with yohimbine at a dose of 5 and 2 mg/kg/day had no effect on the level of sulfane sulfur which remained decreased when compared to control livers (Figure 2B). The level of bound sulfane sulfur, which is a pool of the total sulfane sulfur and includes mainly persulfides and polysulfides, was decreased in the liver of untreated obese animals and obese animals after yohimbine administration. However, the performed statistical analysis revealed a significant decrease in bound sulfane sulfur only in the group of obese animals treated with yohimbine at a dose of 2 mg/kg/day (Figure 2C).

### 2.3. The Effect of HFD and Yohimbine Treatment on the Sulfate Concentration in the Rat Liver

**Figure 3:**
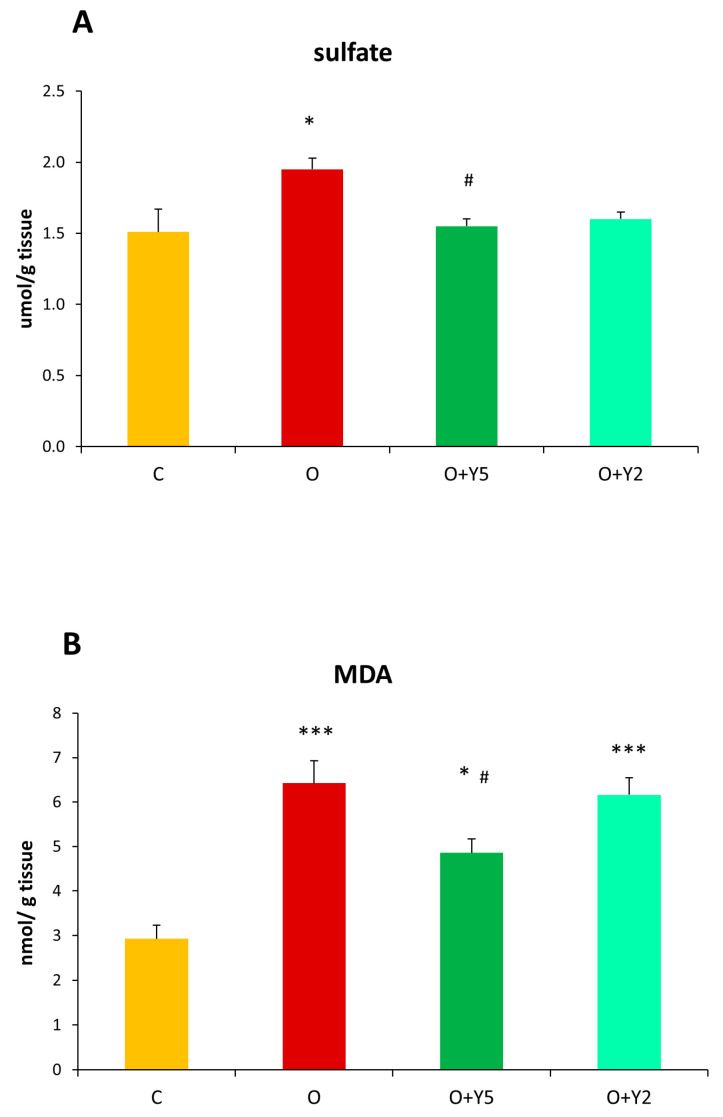
Concentration of sulfates (**A**) and the level of malondialdehyde (MDA) as a measure of lipid peroxidation (**B**) in the livers of rats. C—control rats fed a standard diet; O—rats fed with HFD; O + Y5—rats fed with HFD and then treated intraperitoneally (i.p.) with yohimbine at a dose of 5 mg/kg/day for 30 days; O + Y2—rats fed with HFD and then treated intraperitoneally (i.p.) with yohimbine at a dose of 2 mg/kg/day. Data are presented as the mean ± SEM, *n* = 6 per group. Statistical analysis was performed using a one-way ANOVA; symbols indicate significance of differences according to the Tukey post hoc test, * *p* < 0.05, *** *p* < 0.001 vs. group C; # *p* < 0.05 vs. group O.

The level of sulfates in the liver of obese rats was significantly increased as compared to the level in the liver of control animals (Figure 3A). Treatment with yohimbine at a dose of 5 mg/kg/day significantly decreased the sulfate level to the control value. Yohimbine at a dose of 2 mg/kg/day also reduced the level of sulfates, however, this effect was not statistically significant (Figure 3A).

### 2.4. The Effect of HFD and Yohimbine Treatment on the Level Malonyl Dialdehyde (MDA) as a Marker of Oxidative Stress

The level of MDA was significantly elevated in the liver of rats fed with HFD when compared to the liver of healthy animals. The treatment with yohimbine at a dose of 5 mg/kg/day resulted in a decrease in MDA level in comparison to obese animals without yohimbine treatment, however, the level of MDA was still higher than MDA level in the liver of healthy rats. In contrast, administration of yohimbine at a lower dose (2 mg/kg/day) had no effect on the level of MDA in the liver of obese rats (Figure 3B).

### 2.5. The Effect of HFD and Yohimbine Treatment on the Activity of Enzymes Involved in Reactive Sulfur Species Formation and Transport (CSE, MST, and TST) in the Rat Liver

**Figure 4:**
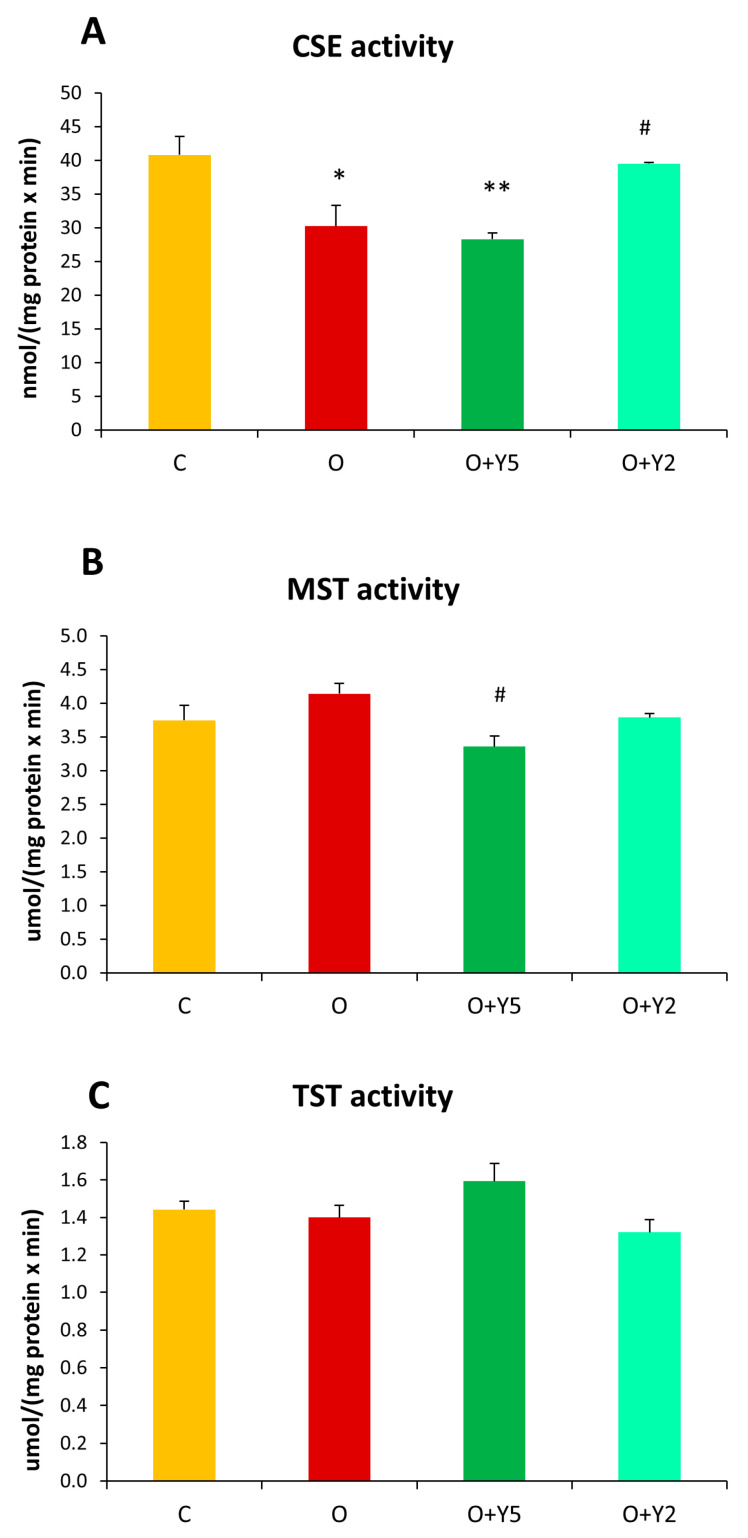
The activity of cystathionine γ-lyase (CSE) (**A**), 3-mercaptopyruvate sulfurtransferase (MST) (**B**), and rhodanese (TST) (**C**) in the livers of rats. C—control rats fed a standard diet; O—rats fed with HFD; O + Y5—rats fed with HFD and treated intraperitoneally (i.p.) with yohimbine at a dose of 5 mg/kg/day for 30 days; O + Y2—rats fed with HFD and treated intraperitoneally (i.p.) with yohimbine at a dose of 2 mg/kg/day. Data are presented as the mean ± SEM, *n* = 6 per group. Statistical analysis was performed using a one-way ANOVA; symbols indicate significance of differences according to the Tukey post hoc test, * *p* < 0.05, ** *p* < 0.01 vs. group C; # *p* < 0.05 vs. group O.

The activity of CSE in the liver of obese rats was significantly reduced compared to the liver of control animals. Treatment with yohimbine at a dose of 5 mg/kg/day did not affect the decreased CSE activity but, interestingly, yohimbine administered at a dose of 2 mg/kg/day increased this activity to the control level (Figure 4A). On the other hand, the activity of MST, slightly but non-significant elevated in the liver of obese rats, was reduced by treatment with yohimbine at a dose of 5 mg/kg/day, while the smaller dose of yohimbine did not affect MST activity (Figure 4B). The hepatic activity of TST was not significantly different in any of the studied obese animals, but its slight non-significant increase could be seen in the group after yohimbine administration at a dose of 5 mg/kg/day (Figure 4C).

### 2.6. The Effect of HFD and Yohimbine Treatment on the Expression of Enzymes Involved in the Formation of Reactive Sulfur Species (CSE, MST, CBS, and TST) in the Rat Liver

**Figure 5:**
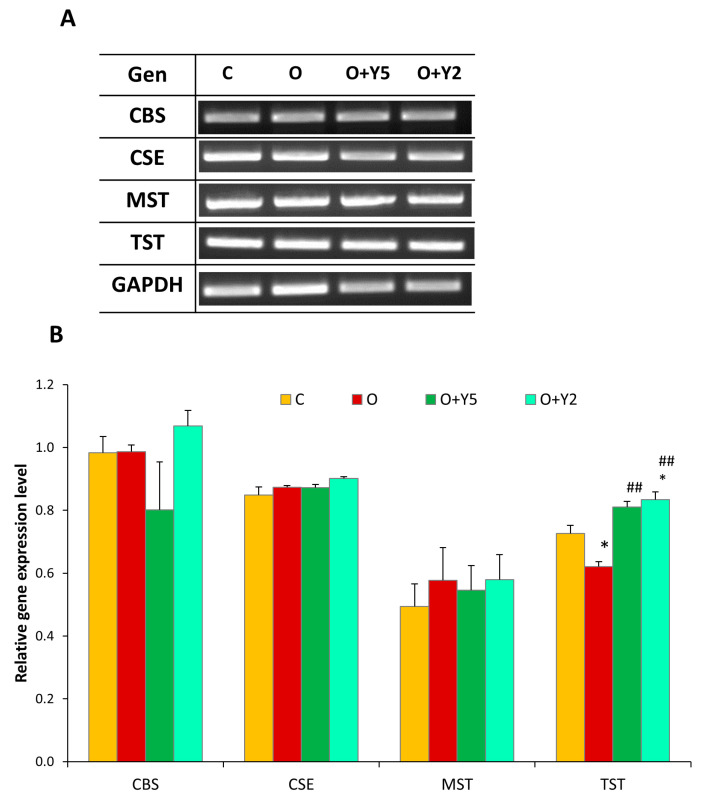
RT-PCR analysis. (**A**) Gene expression in the liver of rats. The results are representative and were chosen from 3 tests. (**B**) The relative expression level of CBS, CSE, MST, and TST in the livers of rats. Densities of bands were normalized using the signal for the GAPDH gene. C—control rats fed a standard diet; O—rats fed with HFD; O + Y5—rats fed with HFD and treated intraperitoneally (i.p.) with yohimbine at a dose of 5 mg/kg/day for 30 days; O + Y2—rats fed with HFD and treated intraperitoneally (i.p.) with yohimbine at a dose of 2 mg/kg/day. Data are presented as the mean ± SEM, *n* = 3 per group. Statistical analysis was performed using a one-way ANOVA; symbols indicate significance of differences according to the Tukey post hoc test, * *p* < 0.05 vs. group C; ## *p* < 0.01 vs. group O.

Gene expression of enzymes involved in reactive sulfur metabolism was another objective of the presented study. Generally, the level of mRNA of the enzymes involved in the synthesis of H_2_S and sulfane sulfur compounds (CSE, MST, and CBS) was unaffected in the liver of HFD-fed animals when compared to healthy, lean rats, and yohimbine did not affect the expression of these enzymes (Figure 5). On the other hand, our study revealed that expression of TST, the enzyme transporting reactive sulfur and forming thiosulfate was diminished in the liver of obese rats, while treatment with yohimbine at both doses (5 mg and 2 mg) increased TST expression (Figure 5).

### 2.7. The Effect of HFD and Yohimbine Treatment on the Level of the Main Low Molecular Weight Thiols (GSH, CSH)

**Figure 6:**
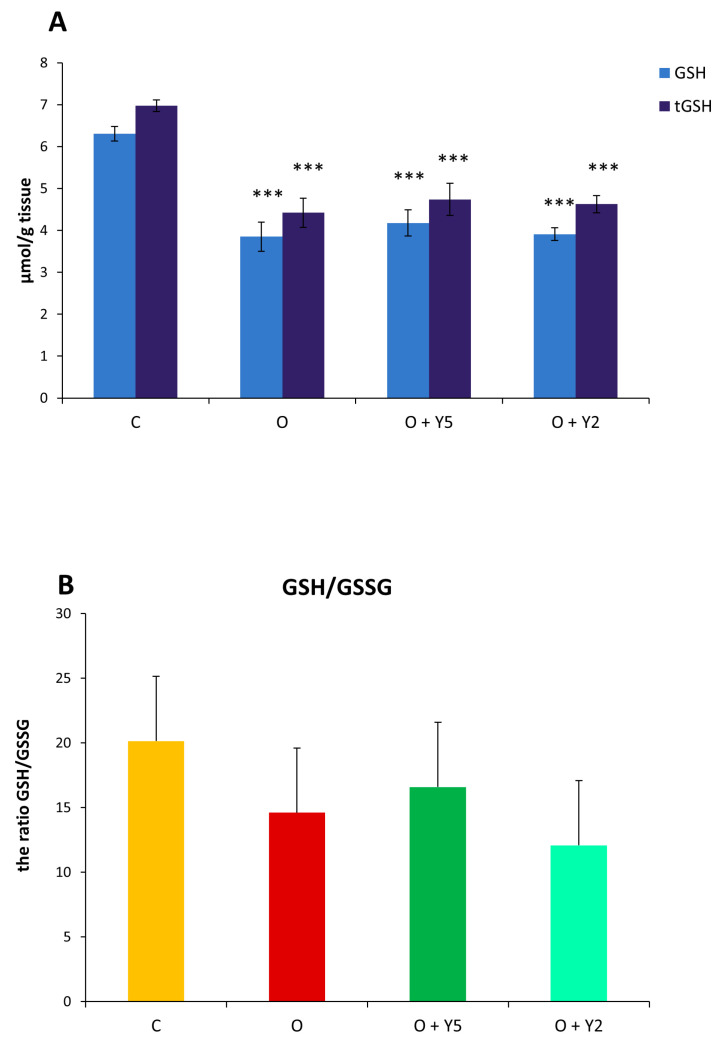
The level of reduced (GSH) and total (tGSH) glutathione (**A**) and the GSH/GSSG ratio (**B**) in the livers of rats. C—control rats fed a standard diet; O—rats fed with HFD; O + Y5—rats fed with HFD and treated intraperitoneally (i.p.) with yohimbine at a dose of 5 mg/kg/day for 30 days; O + Y2—rats fed with HFD and treated intraperitoneally (i.p.) with yohimbine at a dose of 2 mg/kg/day. Data are presented as the mean ± SEM, *n* = 6 per group. Statistical analysis was performed using a one-way ANOVA; symbols indicate significance of differences according to the Tukey post hoc test, *** *p* < 0.001 vs. group C.

The obtained results revealed that HFD-induced obesity caused a significant decrease in the concentration of both forms of glutathione: reduced (GSH) and total (tGSH) (Figure 6A). Treatment of obese rats with yohimbine, especially with a dose of 5 mg resulted in a slight enhancement in GSH and tGSH concentration when compared to the liver of untreated obese rats, but this effect was statistically non-significant. Based on the obtained glutathione concentrations, the GSH/GSSG ratio was calculated for each group of rats and presented in Figure 6B. The statistical analysis did not reveal significant differences between groups, however, a tendency towards a decreased GSH/GSSG ratio in the liver of obese animals was visible and yohimbine did not change it.

**Figure 7:**
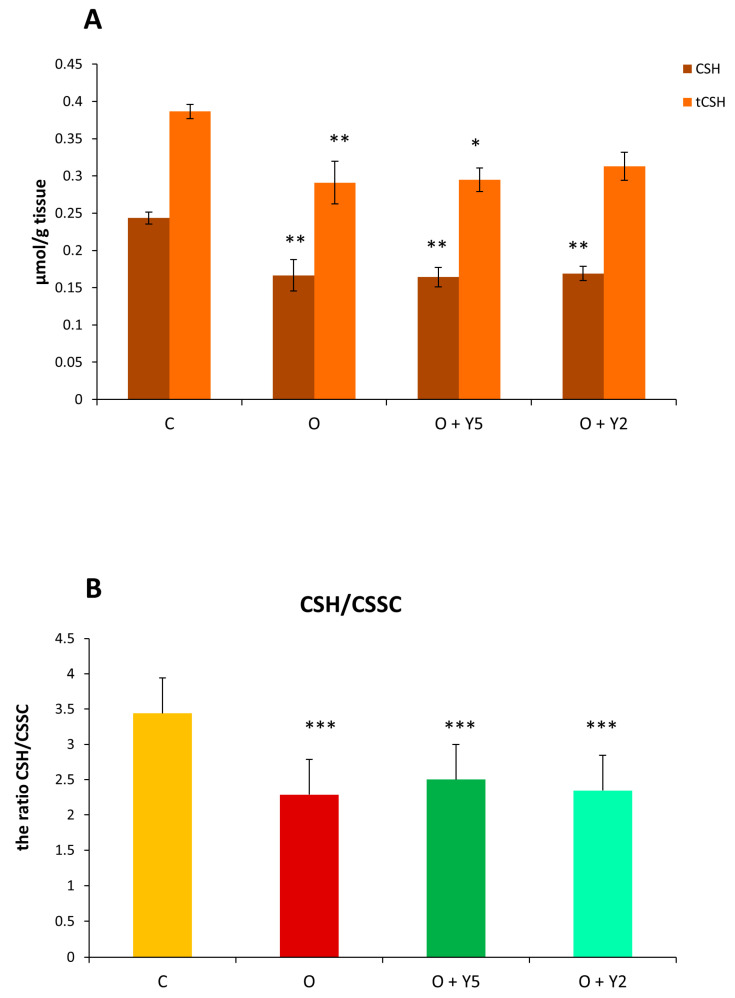
The level of reduced (CSH) and total (tCSH) cysteine (**A**) and the CSH/CSSC ratio (**B**) in the livers of rats. C—control rats fed a standard diet; O—rats fed with HFD; O + Y5—rats fed with HFD and treated intraperitoneally (i.p.) with yohimbine at a dose of 5 mg/kg/day for 30 days; O + Y2—rats fed with HFD and treated intraperitoneally (i.p.) with yohimbine at a dose of 2 mg/kg/day. Data are presented as the mean ± SEM, *n* = 6 per group. Statistical analysis was performed using a one-way ANOVA; symbols indicate significance of differences according to the Tukey post hoc test, * *p* < 0.05, ** *p* < 0.01, *** *p* < 0.001 vs. group C.

The level of reduced cysteine (CSH) was significantly lowered in the liver of obese rats and yohimbine regardless of the used dose did not affect this situation (Figure 7A). In the case of total cysteine (tCSH), its level was also decreased in obese rats’ livers and in the liver of obese rats after yohimbine treatment. The calculated CSH/CSSC ratio was significantly decreased in the liver of obese animals (Figure 7B). In the liver of obese rats treated with yohimbine at a dose of 5 mg, the CSH/CSSC ratio was slightly higher than in the untreated obese group, however, this effect was not significant. It can be concluded that obesity results in a significant drop in the level of glutathione and cysteine, especially their biologically active reduced forms and, in this way, it disrupts the redox status. Yohimbine in the doses used in this study was unable to alleviate these disturbances.

### 2.8. The Effect of HFD and Yohimbine Treatment on the Activity of Glutathione S-Transferase

**Figure 8:**
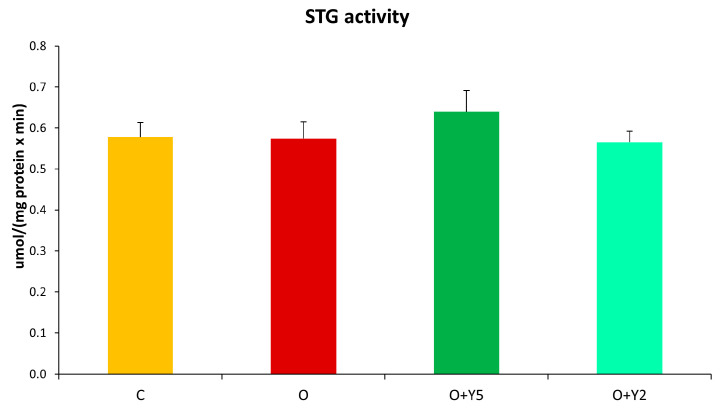
The activity of glutathione-S-transferase (GST) in the livers of rats. C—control rats fed a standard diet; O—rats fed with HFD; O + Y5—rats fed with HFD and treated intraperitoneally (i.p.) with yohimbine at a dose of 5 mg/kg/day for 30 days; O + Y2—rats fed with HFD and treated intraperitoneally (i.p.) with yohimbine at a dose of 2 mg/kg/day. Data are presented as the mean ± SEM, *n* = 6 per group. Statistical analysis was performed using a one-way ANOVA.

Obesity did not affect the activity of GST in the rat liver when compared to control animals. Administration of yohimbine also did not influence GST activity, however, in the group of rats treated with a higher dose of yohimbine (5 mg) a slight but statistically non-significant elevation of GST activity can be observed (Figure 8).

## 3. Discussion

Our study shows that HFD-induced obesity results in many disturbances in the level of low molecular weight thiols and reactive sulfur species in the rat liver. The study showed that treatment of obese animals with yohimbine for 30 days led to a reduction in body weight (Table 1). Moreover, a previous study revealed that yohimbine administrated at a dose of 5 mg caused a reduction of total food intake and intraperitoneal adipose tissue accumulation [19]. In our study, the effectiveness of yohimbine in alleviating disturbances in cysteine metabolism was not impressive, however, the obtained results demonstrated that it restored sulfate concentration and could affect the expression of rhodanese (TST). Moreover, yohimbine at a dose of 5 mg effectively alleviated oxidative stress by lowering the level of MDA in the liver of obese rats.

The link between hepatic disturbances in obese individuals and H_2_S production has been the subject of some previous studies that provided conflicting results. Liu et al. reported the downregulation of CSE in the liver of HFD-induced obese mice, while the expression of CBS was unchanged [20]. In turn, Peh et al. reported diminished expression of CSE and MST in the liver of mice fed with a high-fat diet, while the expression of CBS was elevated compared with control mice [21]. On the other hand, Hwang et al. investigating the effect of a 5-week high-fat diet (HFD) on hepatic CBS and CSE expression and H_2_S level revealed that the CBS and CSE mRNA and protein levels and the level of H_2_S in the liver of HFD-fed mice were significantly elevated compared to control mice [22]. Our results did not reveal changes in the mRNA levels of enzymes responsible for H_2_S synthesis, i.e., CSE, MST, and CBS in the liver of HFD-induced obese rats (Figure 5). It seems that these discrepancies between the obtained results can be connected primarily with differences in the duration of HFD feeding (5–24 weeks) and the feed composition (various fat percentages from 16% to 60% and additional diet components, i.e., cholesterol or cholic acid). The use of different species, mice or rats, might also contribute to the observed differences. In our study, the activity of two main enzymes participating in H_2_S and sulfane sulfur synthesis were also assayed. It should be underlined here that the expression of enzymes does not have to match the activity of enzymes because many proteins can be modified after translation, which affects their activity. Our study revealed that the hepatic activity of CSE was diminished in the liver of obese animals (Figure 4A) despite no change in its expression. Similar results were obtained by Bravo et al. who reported significantly reduced hepatic activity of CSE in the liver of rats fed with HFD [23]. Our results revealed also that the treatment with yohimbine at a dose of 5 mg did not affect CSE activity (Figure 4A). Surprisingly, the treatment with yohimbine at a dose of 2 mg resulted in an increase in CSE activity compared to the liver of obese rats without the yohimbine treatment. In relation to hepatic MST activity, we did not find out significant changes in the liver of HFD-fed rats compared to control animals. The treatment of the obese rats with yohimbine at a dose of 5 mg caused a slight decrease in MST activity (Figure 4B).

Our study did not reveal changes in the level of free H_2_S in the liver of obese rats compared to control animals and yohimbine at both used doses did not affect hepatic free H_2_S levels (Figure 2A). On the other hand, our study clearly showed for the first time a significant decrease in sulfane sulfur level in the liver of rats fed with HFD (Figure 2B). None of the used yohimbine doses affected the sulfane sulfur level. Like the total sulfane sulfur, the level of bound sulfane sulfur consisting mainly of persulfides was also diminished in the liver of HFD-fed rats without and after the treatment with yohimbine, however, in this case, the changes were not significant. In light of these results, it can be concluded that the biological activity of yohimbine causing the loss of fat mass is not related to the production of reactive sulfur species including H_2_S and sulfane sulfur in the rat liver.

The product of lipid peroxidation, malondialdehyde (MDA) is regarded as a biomarker of peroxidative tissue damage. Lipid peroxidation is initiated mainly by reactive oxygen species (ROS) and involves the degradation of polyunsaturated fatty acids (PUFA). ROS detach the hydrogen atom from a PUFA chain transforming fatty acid to free radicals which then generate lipid peroxides. MDA is the well-known end product of lipid peroxides β-oxidation. Our study revealed that the level of MDA was significantly elevated in the liver of HFD-fed rats compared to animals fed a standard diet which indicates lipid peroxidation and suggests that HFD induces oxidative stress in hepatocytes. Similar results were described by other authors previously [16,24,25]. Some of them also reported the decreased activity of SOD in the liver of animals fed with HFD [16,25]. The increased MDA level and diminished activity of SOD were also detected in the serum or plasma of HFD-fed animals [25,26,27]. Our study also revealed a decrease in the hepatic concentration of reduced and total glutathione in HFD-fed rats (Figure 6A). The decrease in GSH level in the liver of animals fed with HFD was also reported by some other researchers [16,28,29]. GSH is the main cellular low molecular weight thiol that can scavenge ROS by oxidizing to glutathione disulfide (GSSG) and in this way, it plays an important antioxidant role. The decrease in GSH level confirms that HFD induces oxidative stress in the liver [16]. However, our study also revealed a diminished concentration of total glutathione (GSH and GSSG) suggesting that in the liver of HFD-fed rats, the synthesis of GSH is inhibited. Similarly to glutathione, both forms of cysteine, reduced and total were diminished in the liver obtained from HFD-fed rats (Figure 7A). The reduced concentration of cysteine, as well as of homocysteine was also reported previously in the liver of mice fed with HFD [30]. As cysteine is the rate-limiting amino acid in the synthesis of GSH, its deficit contributes to a reduction of GSH concentration. On the other hand, cysteine is a substrate for the synthesis of sulfane sulfur; therefore, the decreased cysteine concentration is reflected by a diminished sulfane sulfur level. Cysteine is synthesized from exogenous methionine with the participation of CSE, the activity of which is decreased in the liver of HFD-fed rats, as revealed in our study. The thiol/disulfide ratio is regarded as an indicator of the redox state in the cell. The ratio calculated for glutathione and cysteine showed a decrease in GSH/GSSG and CSH/CSSC ratio in the liver derived from HFD-fed animals, but in the case of the GSH/GSSG ratio these changes were not significant (Figure 6B). These results indicate an impairment of the redox status in the hepatocytes after feeding with HFD which confirms oxidative stress. The decrease in the GSH/GSSG ratio in the liver of HFD-fed animals was also reported by Luo et al. [24] however, other authors did not confirm it. Hwang et al. reported that in the liver of HFD-fed mice, lipid peroxides were elevated while the level of total glutathione remained unchanged [22]. In turn, a study by Moreno-Fernández et al. revealed an increase in hepatic GSH level in the liver of HFD-fed rats despite an increase in MDA level [26], while other authors reported diminished GSH concentration in the liver derived from HFD-fed animals like in our study [16,29].

Our results did not show HFD’s impact on GST activity despite the diminished GSH concentration (Figure 8). Similar results were obtained recently by Santativongchai et al. in the liver of rats fed with HFD for 20 weeks [31]. On the other hand, a previous study by Akbay et al. suggested a decreased activity of GST in the liver of rats after 4 weeks of HFD [32]. It seems that relatively short feeding with HFD lasting 4 weeks results in a decrease in GST activity due to the diminished level of GSH being its substrate. Further, induction of GST expression can be evoked as a compensation mechanism, which seems to be confirmed by a previous study reporting that one of the GST isoenzymes, glutathione S-transferase M2 (GSTM2) was highly upregulated in the liver of mice fed with HFD [33]. As a result of the decreased availability of the substrate and upregulated expression, no difference in the activity of total GST was observed between the liver of HFD-fed rats and control rats. According to our research, the treatment with yohimbine did not affect GST activity (Figure 8).

All our above-described results confirmed that HFD led to oxidative stress in the liver. Our study showed that yohimbine at a dose of 5 mg was able to decrease hepatic MDA level, however, it was still increased when compared to control animals (Figure 3B). The antioxidant potential of yohimbine was poorly studied. Most studies reported its use as an α_2_-adrenoreceptor antagonist to study the therapeutic potential and mechanism of action of some other pharmaceutics, such as dexmedetomidine which is a selective agonist of α_2_-receptors [34,35]. In this aspect, it has been reported that the blockade of α_2_-adrenoreceptor by yohimbine aggravated oxidative stress induced by lipopolysaccharide (LPS) in the liver [30] as well as in the nervous system [34,35], while dexmedetomidine attenuated LPS-induced deleterious effects. On the other hand, Shen et al. reported that yohimbine significantly reduced LPS-induced elevations of MDA in the rat brain, suggesting its antioxidant properties [36].

Our results showed that HFD induced an elevation in hepatic sulfate level suggesting an intensification of aerobic cysteine metabolism. It was confirmed by a previous study that reported elevated levels of taurine, which is also the end product of aerobic cysteine transformation [29] (Figure 1). Interestingly, our study revealed that yohimbine at a dose of 5 mg effectively lowered the level of sulfates to the control value (Figure 3A). It has been demonstrated previously in a liposomal and skeletal muscle model that sulfite radicals are able to initiate lipid oxidation [37]. Thus, it is likely that the increase in lipid peroxidation in the liver of HFD-fed rats is at least partially evoked by elevated production of sulfite radicals. This is all the more likely because the treatment of animals with yohimbine at a dose of 5 mg diminished both lipid peroxidation and sulfate production (Figure 3A,B). Moreover, as revealed by our study, the expression of TST mRNA was decreased in the liver of rats fed with HFD, while yohimbine induced this expression (Figure 5). It may be recalled here that TST is the enzyme that can create thiosulfate utilizing sulfite ions (Figure 1). The activity of TST was also slightly increased in the liver of rats treated with yohimbine at a dose of 5 mg when compared to HFD-fed animals without yohimbine treatment, but it was not significant, probably due to diminished concentration of sulfane sulfur.

Moreover, it has been reported previously that the activity of TST can be inhibited by sulfates, H_2_O_2_, and oxidative stress. On the other hand, TST is activated by GSH and CSH [38,39]. In our study on the liver of obese rats, oxidative stress decreased GSH and CSH, and elevated concentrations of sulfates were detected, and these factors can explain the lack of increase in TST activity in the O +Y5 group despite the observed increase in TST expression. It seems that yohimbine is able to reduce lipid peroxidation and aerobic cysteine metabolism by inducing the expression of TST and raising thiosulfate production but further studies are necessary to confirm this assumption.

**Figure 9:**
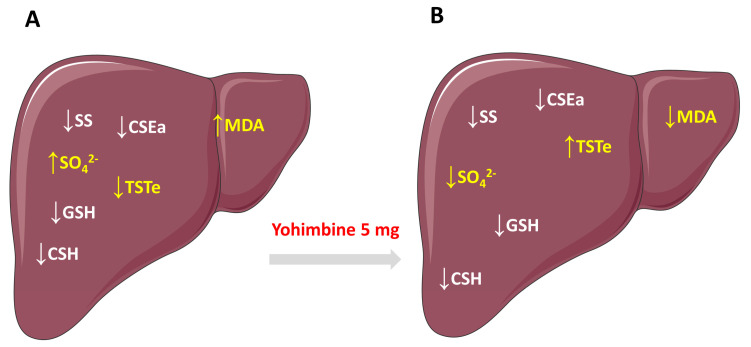
Graphic illustration of the changes in the level of thiols, cysteine catabolism, and lipid peroxidation induced by HFD in the rat liver (**A**) and the effect of yohimbine administered at a dose of 5 mg/kg/day for 30 days (**B**). Arrows indicate: ↑—increase; ↓—decrease. SS—sulfane sulfur, MDA—malonyl dialdehyde; TSTe—expression of rhodanese; CSEa—activity of cystathionine γ—lyase.

Summing up, our results showed that in the liver of rats fed with HFD, cysteine level and its anaerobic catabolism leading to sulfane sulfur compounds were decreased, while sulfate as a product of aerobic cysteine catabolism was elevated. It can be concluded that HFD shifts the balance between anaerobic and anaerobic catabolism of cysteine and induces lipid peroxidation in the rat liver. Our study revealed for the first time that yohimbine administered to HFD-induced obese rats at a dose of 5 mg/kg for 30 days was able to alleviate oxidative stress and diminish the elevated concentration of sulfate, probably by induction of TST expression. This is summarized in Figure 9.

## 4. Materials and Methods

### 4.1. Animals

The experiments were carried out on 8-week-old male Wistar rats (initial body weight 153.6 ± 3.8. The animals were housed in pairs in plastic cages at a constant temperature, exposed to natural light–dark cycle (12/12), and had free access to water and food. All efforts were made to minimize the number and suffering of animals. Experiments were conducted according to the guidelines of the Animal Use and Care Committee of Jagiellonian University and were approved by Local Ethics Committee. (Permission No 54/2012). All animals were divided randomly into four groups, each of which consisted of six rats. Obesity was induced in three groups; one group was the healthy control group.

### 4.2. Obesity Induction

Rats were fed a fatty diet consisting of 40% fat blend (Labofeed B with 40% lard, Morawski, Manufacturer Feed, Poland) for 10 weeks and water was available ad libitum. Control rats were fed a standard diet (Labofeed B) for the same period of time. After 10 weeks, the final body weight was statistically significantly higher when compared to control animals (Table 1). Diet-induced obese rats were randomly divided into three groups (mean body weight in each group was the same) and were treated intraperitoneally (i.p.) with yohimbine at a dose of 2 mg/kg/day (Group O + Y2), yohimbine at a dose of 5 mg/kg/day (Group O + Y5) or with vehicle (distilled water in the same volume as yohimbine, i.e., 0.3 mL; Group O), once daily for 30 days. On the 31st day, animals in all groups were anesthetized with thiopental (70 mg/kg) by intraperitoneal injection and the livers were isolated, frozen in liquid nitrogen, and stored at −80 °C for further biochemical assays.

### 4.3. Chemicals

Yohimbine was obtained from Tocris (Bristol, UK), and thiopental from Sandoz International, (France). Potassium cyanide (KCN), potassium thiocyanate (KSCN), p-phenylenediamine, zinc acetate, thionine, dithiothreitol (DTT), pyridoxal phosphate monohydrate (PLP), L-homoserine, sodium thiosulfate (Na_2_S_2_O_3_), 1-chloro-2,4-dinitrobenzene (CDNB), gelatin, glycine-glycine (Gly-Gly), glutathione reduced form (GSH), lactic dehydrogenase (LDH), L-glutamyl-3-carboxy-4-nitroanilide, 3-mercaptopyruvate (3-MP), 3-methyl-2-benzothiazolinone hydrazine hydrochloride monohydrate (MBTH), N-ethylmaleimide (NEM), β-nicotinamide adenine dinucleotide reduced form (NADH), thiobarbituric acid (TBA) and trichloroacetic acid (TCA) were provided by Sigma-Aldrich Chemical Company (St. Louis, MO, USA). Acetic acid, ammonia (NH_3_), barium chloride (BaCl_2_), copper sulfate (CuSO_4_), Folin–Ciocalteu phenol reagent, formaldehyde, ferric chloride (FeCl_3_), hydrochloric acid (HCl), iron nitrate (Fe(NO_3_)_3_), magnesium chloride (MgCl_2_), nitric acid (HNO_3_), potassium dihydrogen phosphate (KH_2_PO_4_), perchloric acid (HClO_4_), sodium carbonate (Na_2_CO_3_), sodium hydroxide (NaOH), sodium sulfite (Na_2_SO_3_) were obtained from the Polish Chemical Reagent Company (P.O.Ch, Gliwice, Poland). HPLC-grade acetonitrile (ACN) and perchloric acid (PCA) were from J.T. Baker (Deventer, The Netherlands). 2-Chloro-1-methylquinolinium tetrafluoroborate (CMQT) was prepared according to the procedure described by Bald and Głowacki [40] in the Department of Environmental Chemistry, University of Łódź (Łódź, Poland).

### 4.4. Preparation of Tissue Homogenates

All experimental procedures involved in the preparation of tissue homogenates were carried out at 4 °C. The frozen liver tissue samples were weighed and homogenized using an IKA-ULTRATURRAX T8 homogenizer for biochemical assays at a ratio of 1 g of tissue to 4 mL of 0.1 M phosphate buffer, pH 7.4, while for HPLC analysis at a ratio of 1 g of tissue to 9 mL of 0.2 M phosphate buffer pH 8.0. The obtained homogenate was centrifuged at 2000× *g* for 5 min and the supernatant was used for biochemical assays.

### 4.5. Biochemical Assays

The total level of sulfane sulfur was determined by the method of Wood [41], while the level of free sulfide (H_2_S) was determined using a modified method of Shen et al. [42] with fluorometric detection. The level of sulfate was estimated with the sulfate assay kit (Sigma) according to the manufacturer’s instructions. In this method, inorganic sulfate is precipitated by a reaction with barium sulfate in polyethylene glycol for stabilization of turbidity. This method was modified by using gelatin solution instead of polyethylene glycol. The level of sulfide released by reduction with dithiothreitol (DTT) was determined by the modified method of Ogasawara et al. [43] and the level of bound sulfane sulfur including mainly persulfides and polysulfides was obtained after subtracting the level of free sulfide. The activity of CSE was assayed by the modified method of Matsuo and Greenberg [44]. L-homoserine was used as a substrate and α-ketobutyric acid formed in this reaction was assayed according to the method of Soda [45]. The activity of MST was determined by the method of Valentine and Frankenfeld [46], while TST activity was assayed according to Sörbo’s method [47]. Estimation of the GST activity was assayed as described previously [48]. The level of MDA was assayed according to Ohkawa [49], while the protein concentration was by the method of Lowry et al. [50].

### 4.6. High-Performance Liquid Chromatography (HPLC) Analysis

The levels of reduced and total thiols (glutathione and cysteine) were measured by HPLC after precolumn derivatization with 2-chloro-l-methylquinolinium tetrafluoroborate (CMQT) and with ultraviolet detection [51]. The analysis was performed using an HPLC system (Shimadzu Duisburg, Germany) consisting of two high-pressure pumps LC 10AT vp, a degasser DGU-14 A, an autosampler SIL-10ADvp, a column thermostatic oven CTO-10 Asvp and a diode detector SPD-M10Avp. The entire HPLC system was under the control of Shimadzu’s Lab solution software. The samples were separated using a Zorbax Eclipse XDB-18 column (4.6 × 250 mm, 5 μm) from Agilent (Santa Clara, CA, USA), protected by a guard column with the same packing. The temperature was 25 °C, the flow rate was 1 mL/min, and detector wavelength was 350 nm. The elution profile was as follows: 0–4 min, 12% B; 4–7 min, 12–40% B; 7–8 min, 40% B; 8–10 min 40–12% B. Elution solvent A was 0.05 M TCA adjusted to pH 3.2 with lithium hydroxide.

#### 4.6.1. Reduced Thiols

For determination of reduced thiols, 200 μL of tissue homogenate was mixed with 15 μL of 0.2 M phosphate buffer, pH 8.2 and 20 μL of 0.1 M CMQT dissolved in distilled water, and the mixture was incubated at room temperature for 5 min. After derivatization, the mixture was acidified with 30 μL of 3 M perchloric acid (PCA) followed by centrifugation. An amount of 20 μL of the obtained supernatant was subjected to HPLC analysis.

#### 4.6.2. Total Thiols

For determination of the total thiols, 200 μL of tissue homogenate was mixed with 15 μL of 0.25 M TCEP (dissolved in 0.2 M phosphate buffer, pH 8.2 and adjusted to pH 7–8 with 2.5 M NaOH) and the mixture was incubated at a room temperature for 15 min to reduce disulfide bonds. Next, 20 μL of 0.1 M CMQT was added and the procedure was continued as in the case of reduced thiols. After centrifugation, 20 μL of the supernatant was subjected to HPLC analysis.

#### 4.6.3. Oxidized Thiols

The levels of oxidized thiols were estimated by subtracting the level of the reduced thiols from the pool of total thiols and dividing by two (one mol of oxidized thiol consists of two reduced molecules).

### 4.7. Enzyme Expression

#### 4.7.1. Isolation of Total RNA

Total RNA was extracted from the tissues using TRI reagent (Sigma-Aldrich, Darmstadt, Germany), according to the protocol provided by the manufacturer. The extracted RNA was suspended in ribonuclease-free water and quantified by measuring the absorbance at 260 nm. After the isolation procedure, every time, the purity of the obtained RNA was checked by estimating the A260 nm/A280 nm ratio. The integrity of the obtained RNA was confirmed by the separation of the 28S and 18S rRNA bands in 2.0% agarose gel electrophoresis. The RNA solutions were stored at −80 °C until further studies were performed.

#### 4.7.2. Reverse Transcription of RNA

Total RNA from particular tissues was reverse-transcribed using the GoScriptTM Reverse Transcriptase Kit according to the manufacturer’s protocol (Promega, Madison, WI, USA). For reverse transcription (RT), 2 µg of total RNA was mixed with 1 µL Oligo d(T)15 primer (0.5 µg/µL) and water pretreated with diethylpyrocarbonate (DEPC-H_2_O), and the mixture was incubated for 5 min at 70 °C. After preincubation, the samples were placed on ice and other components were added to the mixture: 4 µL 5× concentrated RT buffer (250 mM Tris-HCl, 250 mM KCl, 20 mM MgCl_2_, 50 mM DTT, pH 8.3 at 25 °C), 2 µL deoxyribonucleotide triphosphates (dNTPs, 10 mM) and 1 µL RNase inhibitor (20 U/µL) and 1 µL GoScriptTM Reverse Transcriptase (160 U/µL) in a total volume of 20 µL. After incubation at 25 °C for 5 min, the mixture was incubated for 60 min at 42 °C, and then for the final 10 min at 70 °C. If necessary, the solutions of complementary DNA (cDNA) were stored at −20 °C.

#### 4.7.3. Polymerase Chain Reaction (PCR)

The expression of four genes involved in reactive sulfur species metabolism (CSE, MST, CBS, and TST) was analyzed by PCR using the MiniOpticonTM System (Bio-Rad, Hercules, CA, USA). Glyceraldehyde 3-phosphate dehydrogenase (GAPDH) was used as a reference gene. Amplification of cDNA was run in a 25 µL reaction volume that contained the following constituents: 2 µL of the synthesized cDNA, 10 µM of each of gene-specific primer pair (Table 2), 2 U/µL Taq DNA polymerase in 10 mM buffer Tris-HCl, pH 8.8 (supplemented with 1.5 mM MgCl_2_, 50 mM KCl, 0.1% Triton X-100), 10 mM of each dNTPs and DEPC-H_2_O. The PCR cycling conditions for all selected genes were: 94 °C for 5 min, 26 cycles (for MST, CBS, and TST) or 24 cycles (for CSE and GAPDH) of amplification (94 °C for 30 s, 62 °C for 30 s, and 72 °C for 2 min), and a final extension at 72 °C for 8 min. The PCR reaction conditions for these five genes were established and optimized specifically to address the needs of the present study. In each case, a similar reaction was also performed in the mixture without DNA (negative control) in order to confirm the specificity of the obtained reaction products. All amplification reactions were performed at least three times to ensure the reproducibility of results. All PCR products were analyzed by electrophoresis on a 2.0% agarose gel stained with ethidium bromide and directly visualized under UV light and photographed (ChemiDocTM MP Imaging system with Image Lab Software, version 6.0, Bio-Rad).

### 4.8. Statistical Analysis

The results are presented as the mean values ± standard error of the mean (SEM). For biochemical assays, the mean was calculated from all tissues of animals belonging to a given group, while for enzyme expression the mean was estimated from three determinations. A one-way analysis of variance (ANOVA) followed (if significant) by Tukey test was used for statistical analysis of the data. A *p* value < 0.05 was considered statistically significant.

## Figures and Tables

**Table 1:** Changes in rats’ body weight during obesity induction and during treatment.

Changes in Body Weight During 10 Weeks of Obesity Induction (g)		Changes in Body Weight During 30 Days of Treatment (g)			
C	HFD (O)	C	O	O + Y5	O + Y2
206.7 ± 3.5	256.8 *** ± 4.8	38.7 ± 1.6	57.6 * ± 4.3	19.4 ^###^ ± 6.1	37.0 ^#^ ± 8.3
*n* = 6	*n* = 18	*n* = 6	*n* = 6	*n* = 6	*n* = 6

Comparisons versus control (*) during obesity induction were performed by t-Student test. Comparisons during treatment were performed by one-way ANOVA with Tukey post hoc test. Significant differences versus control * *p* < 0.05, *** *p* < 0.001, and versus the obesity group ^#^ *p* < 0.05, ^###^ *p* < 0.001.

**Table 2:** Primer sequences used for determination of target genes by PCR.

Target Gene	No. in NCBI Database	Primers (5′→3′)	Location in Gene	Product Size [bp]	Ref.
CSE	NM_017074.1	F: CCGACGAGGAATTGCTTGGA R: ACATCACTGTGGCCGTTCAT	203–666	464	[52]
MST	NM_138843.1	F: GTATCTGCTCAGTGGGTGGC R: CAGGGATGTGTCCAGGTTCG	240–828	589	
CBS	NM_012522.2	F: ATGGATGCTGCAGAAAGGCT R: AGGTGGATCGGCTTGAACTG	1302–1607	306	
TST	NM_012808.1	F: ACATCCGTGGCTCTGTCAAC R: TCGTAAACAGCCACATCGGG	642–830	208	[52]
GAPDH	NM_017008.4	F: AGTGCCAGCCTCGTCTCATA R: GATGGTGATGGGTTTCCCGT	50–297	248	

## Data Availability

Data sharing not applicable.

## Sample Availability

Samples of the compounds are not available from the authors.
